# Adolescents’ pain-related ontogeny shares a neural basis with adults’ chronic pain in basothalamo-cortical organization

**DOI:** 10.1016/j.isci.2024.108954

**Published:** 2024-01-17

**Authors:** Nils Jannik Heukamp, Tobias Banaschewski, Arun L.W. Bokde, Sylvane Desrivières, Antoine Grigis, Hugh Garavan, Penny Gowland, Andreas Heinz, Mina Kandić, Rüdiger Brühl, Jean-Luc Martinot, Marie-Laure Paillère Martinot, Eric Artiges, Dimitri Papadopoulos Orfanos, Herve Lemaitre, Martin Löffler, Luise Poustka, Sarah Hohmann, Sabina Millenet, Juliane H. Fröhner, Michael N. Smolka, Katrin Usai, Nilakshi Vaidya, Henrik Walter, Robert Whelan, Gunter Schumann, Herta Flor, Frauke Nees, IMAGEN Consortium

**Affiliations:** 1 Institute of Medical Psychology and Medical Sociology, University Medical Center Schleswig-Holstein, Kiel University, Kiel, Germany; 2 Department of Child and Adolescent Psychiatry and Psychotherapy, Central Institute of Mental Health, Medical Faculty Mannheim, Heidelberg University, Square J5, 68159 Mannheim, Germany; 3 Discipline of Psychiatry, School of Medicine and Trinity College Institute of Neuroscience, Trinity College Dublin, Dublin, Ireland; 4 Centre for Population Neuroscience and Precision Medicine (PONS), Institute of Psychiatry, Psychology & Neuroscience, SGDP Centre, King’s College London, London, UK; 5 NeuroSpin, CEA, Université Paris-Saclay, 91191 Gif-sur-Yvette, France; 6 Departments of Psychiatry and Psychology, University of Vermont, Burlington, Vermont 05405, USA; 7 Sir Peter Mansfield Imaging Centre School of Physics and Astronomy, University of Nottingham, University Park, Nottingham, UK; 8 Department of Psychiatry and Psychotherapy CCM, Charité – Universitätsmedizin Berlin, Corporate Member of Freie Universität Berlin, Humboldt-Universität zu Berlin, and Berlin Institute of Health, Berlin, Germany; 9 Institute of Cognitive and Clinical Neuroscience, Central Institute of Mental Health, Medical Faculty Mannheim, Heidelberg University, Square J5, Mannheim, Germany; 10 Physikalisch-Technische Bundesanstalt (PTB), Braunschweig, Berlin, Germany; 11 Institut National de la Santé et de la Recherche Médicale, INSERM U A10 "Trajectoires développementales en psychiatrie", Université Paris-Saclay, Ecole Normale supérieure Paris-Saclay, CNRS, Centre Borelli, Gif-sur-Yvette, France; 12 AP-HP, Sorbonne Université, Department of Child and Adolescent Psychiatry, Pitié-Salpêtrière Hospital, Paris, France; 13 Psychiatry Department, EPS Barthélémy Durand, Etampes, France; 14 Institut des Maladies Neurodégénératives, UMR 5293, CNRS, CEA, Université de Bordeaux, 33076 Bordeaux, France; 15 Clinical Psychology, Department of Experimental Psychology, Heinrich Heine University Düsseldorf, Düsseldorf, Germany; 16 Integrative Spinal Research Group, Department of Chiropractic Medicine, University Hospital Balgrist, University of Zurich, Zurich, Switzerland; 17 Department of Child and Adolescent Psychiatry and Psychotherapy, University Medical Centre Göttingen, von-Siebold-Str. 5, 37075 Göttingen, Germany; 18 Department of Psychiatry and Neuroimaging Center, Technische Universität Dresden, Dresden, Germany; 19 Centre for Population Neuroscience and Stratified Medicine (PONS), Department of Psychiatry and Neuroscience, Charité Universitätsmedizin, Berlin, Germany; 20 School of Psychology and Global Brain Health Institute, Trinity College Dublin, Berlin, Ireland; 21 Centre for Population Neuroscience and Precision Medicine (PONS), Institute for Science and Technology of Brain-inspired Intelligence (ISTBI), Fudan University, Shanghai, China; 22 Department of Psychology, School of Social Sciences, University of Mannheim, 68131 Mannheim, Germany

**Keywords:** neuroscience, clinical neuroscience, sensory neuroscience

## Abstract

During late adolescence, the brain undergoes ontogenic organization altering subcortical-cortical circuitry. This includes regions implicated in pain chronicity, and thus alterations in the adolescent ontogenic organization could predispose to pain chronicity in adulthood - however, evidence is lacking. Using resting-state functional magnetic resonance imaging from a large European longitudinal adolescent cohort and an adult cohort with and without chronic pain, we examined links between painful symptoms and brain connectivity. During late adolescence, thalamo-, caudate-, and red nucleus-cortical connectivity were positively and subthalamo-cortical connectivity negatively associated with painful symptoms. Thalamo-cortical connectivity, but also subthalamo-cortical connectivity, was increased in adults with chronic pain compared to healthy controls. Our results indicate a shared basis in basothalamo-cortical circuitries between adolescent painful symptomatology and adult pain chronicity, with the subthalamic pathway being differentially involved, potentially due to a hyperconnected thalamo-cortical pathway in chronic pain and ontogeny-driven organization. This can inform neuromodulation-based prevention and early intervention.

## Highlights

• Adolescent ontogenic basothalamo-cortical changes and painful symptoms correlate
• Basothalamo-cortical brain connectivity is altered in adult chronic pain
• The overlapping neural basis indicates complex brain-pain trajectories
• Late adolescence may be a sensitive period of pain-related brain changes

## Graphical abstract

**Figure:**
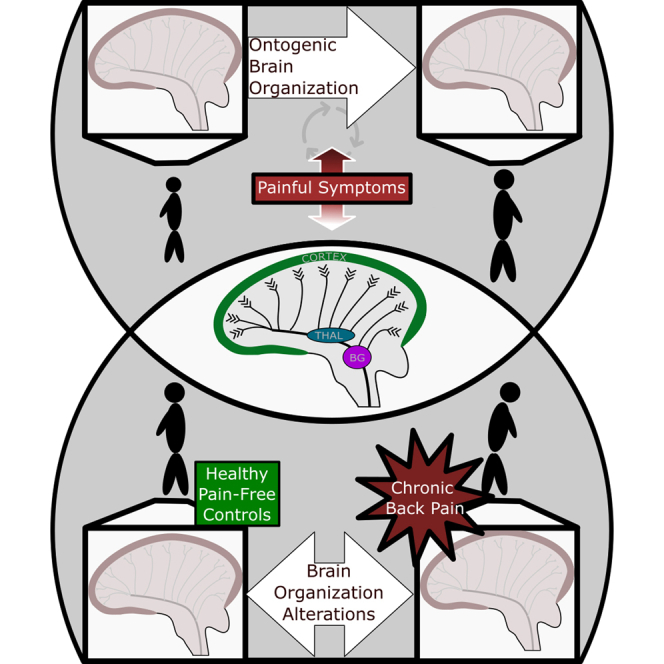

## Introduction

Adolescence is characterized by the ontogenic organization of the brain that underly imbalance in emotion, cognition, and behavior.^1^^,^^2^^,^^3^ While this is a normal developmental process, some brain-behaviour alterations have been shown to increase the risk for mental disorders^4^ and could similarly also represent risk factor for chronic pain. There is evidence that specifically prefrontal, somatosensory, limbic, and basal ganglia circuitries, and more generally subcortical-cortical pathways are associated with chronic pain.^5^^,^^6^^,^^7^^,^^8^ However, current knowledge is based on functional brain alterations and their association with pain in adult populations, either spanning the transition from subacute to chronic pain^7^^,^^8^^,^^9^^,^^10^ or focusing on the chronic condition.^11^^,^^12^^,^^13^^,^^14^ Data on sensitive periods in the early life, such as during adolescence, is scarce. In one of our previous studies, we observed alterations in the periaqueductal gray and striatum during a reward-related monetary incentive delay task at age 14, together with genetic expressions in the opioid system, as a significant predictor for painful symptoms at age 16.^15^ Other studies reported altered neural responses to pain in the posterior cingulate cortex in adolescents with compared to those without chronic pain,^16^ and showed reduced resting-state connectivity in pain-related sensory brain regions being associated with increased pain thresholds in older compared to young individuals.^17^ Decreased activity in the control and salience network as well as increased activity in default mode network was also found to be related to altered reward and emotional and cognitive processing in adolescents with a high familial risk of chronic pain.^18^

While these findings provided first evidence on brain representations of painful symptoms as well as chronicity of pain during adolescence, the role of ontogenic brain organization for pain symptomatology in adolescence is still not clear. Therefore, in the present study, we assessed resting-state brain connectivity changes during late adolescence in 690 individuals from a large European longitudinal study using two time points (TP1, TP2, ⌀(age)TP1 = 18.4(0.70), ⌀(age)TP1 = 22.0(0.68)) and associated these with painful symptoms in late adolescence. This allows us to identify potential ontogenic brain correlates of early pain symptoms in a sensitive period in which pain problems just start to develop. To test for the specificity of the identified brain patterns as a potential correlate of pain chronicity later in life, in terms of risk for the development of chronic pain and/or adaption processes related to pain chronification, we additionally analyzed resting-state brain connectivity in an independent sample of 29 adult patients with chronic pain (⌀(age) = 38.7(15.8)) as well as 29 age-matched healthy pain-free controls (⌀(age) = 35.7(14.7)) using data from Collaborative Research Center Initiative.

## Results

### Basothalamo-Cortical Brain Connectivity Change is associated with painful symptoms in late adolescence

**Figure 1:**
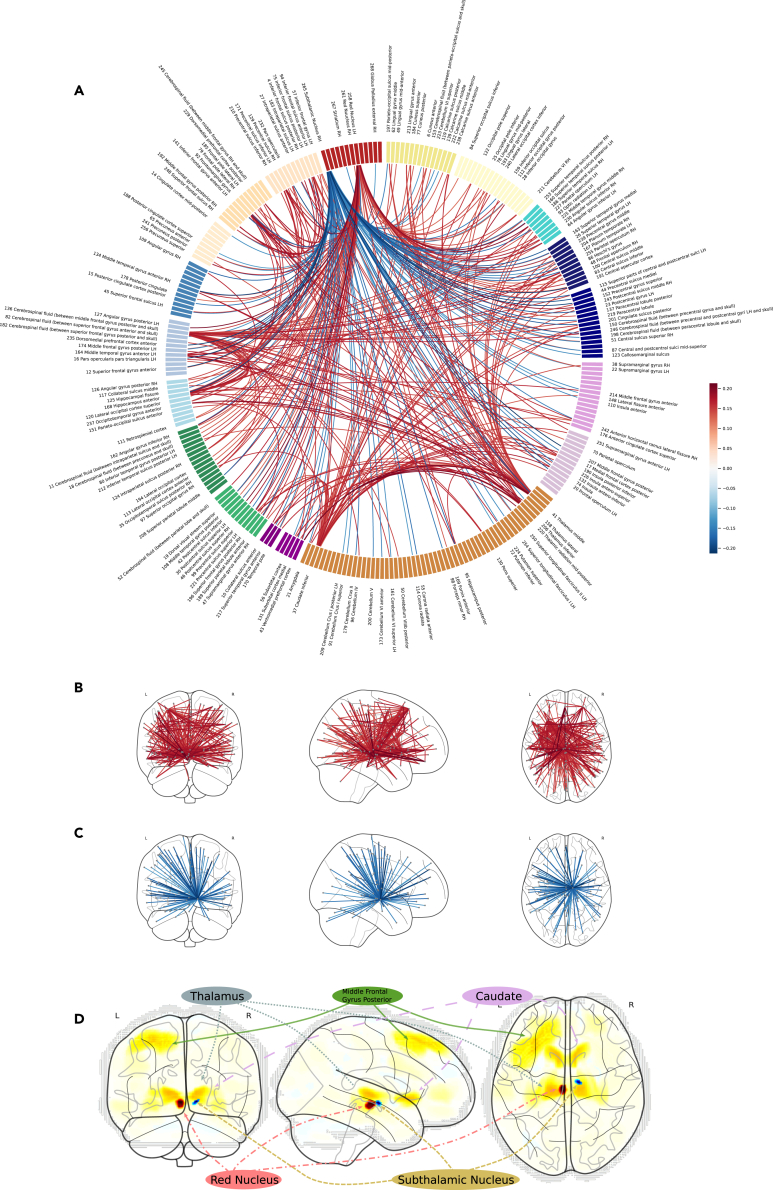
Basothalamo-Cortical Brain Connectivity Change is Associated with Painful Symptoms in Late Adolescence (A) Significant associations (β, p_adj_ ≤0.05) of ΔrsFCs with painful symptoms. Ordered network wise according to Yeo-16-network-parcellation (2011) Control networks (beige), default-mode networks (light blue), dorsal attention networks (green), limbic networks (purple), salience ventral attention network (pink), somatomotor networks (dark blue), temporal-parietal network (turquoise), visceral central networks (light yellow), no network (orange), basal ganglia parcellation (red). (B) ΔrsFCs with significant positive associations to painful symptoms colored according to effect strength projected on MNI-Space. (C) ΔrsFCs with significant negative associations to painful symptoms colored according to effect strength projected on MNI-Space. (D) Hubs of ΔrsFCs associated with painful symptoms at TP2. Plotted is the product of each probabilistic ROI map with the ROIs number of positively associated ΔrsFCs subtracted by the number of negatively associated ΔrsFCs. Red means the hub’s connectivities are mostly positively associated, blue negatively associated with painful symptoms.

**Figure 2:**
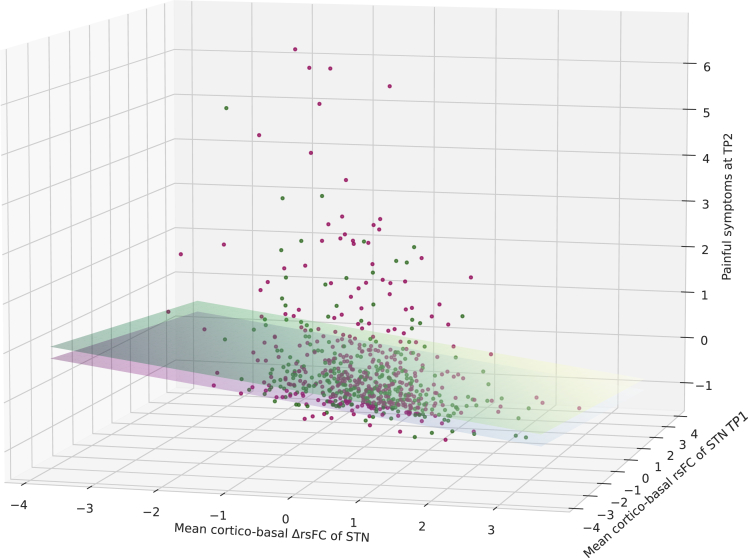
Change of cortical integration of the STN is associated with painful symptoms in late adolescence Scatterplot of z-scaled mean rsFC of the STN at TP1 (x) its difference to TP2 (y) and painful symptoms at TP2 (z) colored for sex (F: green, M: purple). Prediction of the regression model are drawn as plane separated by sex (yellow green: female, blue purple: male).

First, we tested the association of painful symptoms with ontogenic brain organization in the course of late adolescence. Therefore, we examined the individual change of resting state functional connectivity (rsFC) between TP1 and TP2 (ΔrsFCs) and used every ΔrsFCs separately to correlate with painful symptom score at TP2 while controlling for the corresponding rsFC at TP1 and sex using connectivity-wise ordinary least-square regressions (df = 686). We found n (p_adj_ ≤0.05) = 346 ΔrsFC to be significantly associated with painful symptoms at TP2 (Figure 2). The pattern of brain connectivities, that were associated with painful symptoms, included ΔrsFCs of the right Subthalamic Nucleus (STN) with regions of particularly the somatomotor, salience and limbic networks, that were negatively associated with painful symptoms at TP2 (n(p_adj_ ≤0.05) = 97) as well as ΔrsFCs of the left Red Nucleus (RN) with regions of particularly the dorsal attention and somatomotor network (n(p_adj_ ≤0.05) = 84), ΔrsFCs of the middle and inferior thalamus (n(p_adj_ ≤0.05) = 42, n(p_adj_ ≤0.05) = 20) and the caudate inferior (n(p_adj_ ≤0.05) = 38) with particularly regions of the limbic network as well as multiple cortical areas. Except for the ΔrsFCs of the STN all regions ΔrsFCs were positively associated with painful symptoms at TP2 (Figure 1, Table 1). The rsFC at TP1 which were added as confounder showed n(β_α≤0.05_) = 17 significant associations with painful symptoms at TP2 of which 14 were rsFCs of the genu of the callosal body. For detailed network-wise accumulated and weighted results see Table 1, for ROI-wise see Table S1.

**Table 1:** Frequency of significantly associated ΔrsFC changes of subcortical ROIs with ROIs of the resting-state networks

Resting-State-Network	Subcortical ROI					
	caudate inferior	thalamus middle	hippocampus anterior	thalamus inferior	red nucleus LH	subthalamic nucleus RH
control	–	0.185	0.222	0.074	0.259	0.185
default-mode	–	0.125	0.250	0.094	0.375	0.375
dorsal attention	0.133	0.100	0.033	0.033	0.733	0.300
limbic	0.429	0.571	–	0.571	0.286	0.571
salience ventral attention	0.357	0.071	0.071	0.036	0.179	0.571
somatomotor	0.250	0.214	0.035	0.036	0.536	0.750
visual	0.317	0.219	–	0.170	0.366	0.171

Frequency calculated as number of significant connectivities to one specific network relative to the total amount of connectivities to that network. LH = Left Hemisphere, RH = Right Hemisphere.

Additionally, due to the significant and prominent role of the ΔrsFCs of the STN in the pattern of brain connectivities associated with painful symptoms at TP2 we additionally analyzed the association of painful symptoms with the ontogenic cortical integration of the STN in the connectome. Ontogenic cortical Integration and the corresponding level at TP1 was examined as the mean cortical ΔrsFCs of the STN. We then applied a model equivalent to that for the association of painful symptoms at TP2 by individual ΔrsFCs with the mean cortical ΔrsFC of the STN while adding the mean cortical rsFC and sex as covariates. This model was significant ($R^{2}$ = 0.03, *F*(686) = 8.198, p < 0.001, Figure 2, Table 2), with the mean cortical ΔrsFCs of the STN being negatively associated with painful symptoms at TP2 (*β* = −0.18, p < 0.001, *t*(689) = -3.514), while the mean cortical rsFC of the STN at TP1 was not significantly associated with painful symptoms at TP2 (*β* = −0.0744, p = 0.146, *t*(689) = -1.454). Sex (*β* = −0.241, p = 0.002, *t*(689) = 3.173) however, was significantly associated with painful symptoms at TP2 and the intercept was found significant (*β* = 0.102, p = 0.04, *t*(689) = 2.062). Additionally, we computed supplemental receiver operating characteristic curves and area under the curve for different percentile thresholds (see Figure S1).

**Table 2:** Regression Model of the association of mean cortical connectivity change of the bilateral STN with painful symptoms

	*β*	*std*	*t*	*P>|t|*	*[0.025*	*0.975]*
Mean ΔrsFC of the STN	−0.1797	0.051	−3.514	0.001	−0.280	−0.079
Mean rsFC of the STN at TP1	−0.0744	0.051	−1.454	0.146	−0.175	0.026
sex	−0.2414	0.076	−3.173	0.002	−0.391	−0.092
intercept	0.1018	0.049	2.062	0.040	0.005	0.199

### Basothalamo-Cortical Brain Connectivity Alterations in Adult Chronic Pain

**Figure 3:**
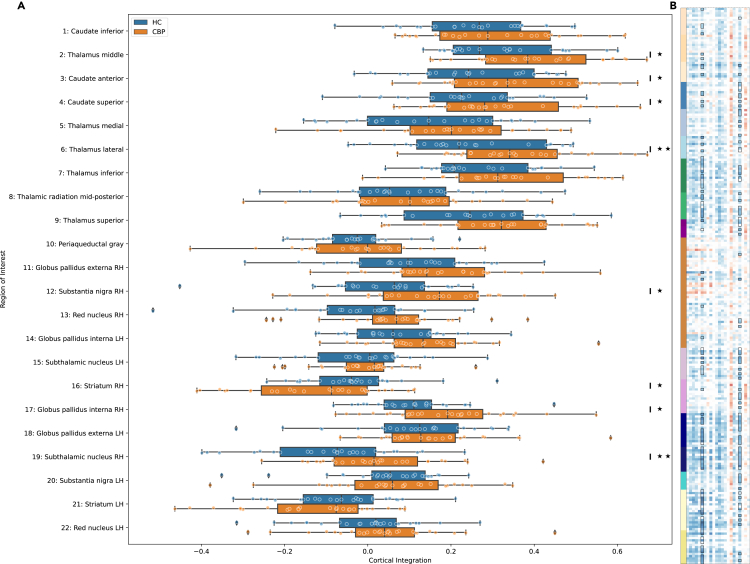
Basothalamo-Cortical Brain Connectivity Alterations in Adult Chronic Pain (A) Comparison of cortical integration between HC and CBP for noticeable ROIs from the main analysis and closely related areas. Cortical integration is significantly increased in multiple ROI of the thalamus as well as those of the right (hyper-) direct pathway. The center lines of the boxplots denotes the median, the boxes the 25th and 75th percentiles and the whiskers the median ± 1.5 interquartile range. ∗ p_adj_ <0.05, ∗∗ p_adj_ <0.01. (B) Connection-wise comparison between HC and SBP for all connections of the same ROIs. *t*-values are plotted. Significance is represented uncorrected via a white overlay (p ≥0.1 opaque, p ≤0.05 transparent, uncorrected) and corrected via black frames (p_adj_ ≤0.05). Columns ordered from left to right according to ROI-number in (A). Colored y axis represents network according to Yeo-16-network-parcellation (2011) Control networks (beige), default-mode networks (light blue), dorsal attention networks (green), limbic networks (purple), salience ventral attention network (pink), somatomotor networks (dark blue), temporal-parietal network (turquoise), visceral central networks (light yellow), no network (orange), basal ganglia parcellation (red).

Finally, to test whether the brain circuits involved in painful symptoms in late adolescence overlap with brain circuits alterations in chronic pain in adulthood, we analyzed whether the pattern of brain connectivities associated with painful symptoms in late adolescence also show difference between patients with chronic pain compared to healthy controls. Therefore, we tested for differences of rsFC of the basal ganglia, thalamus and caudate regions using an ANOVA with sex as additional variable for control. The STN showed n(p_adj_ ≤0.05) = 95 and the thalamus lateral n(p_adj_ ≤0.05) = 105 showed significant differences in rsFC (Figure 3B). Additionally, we tested for alterations in the cortical integration of the right STN like in the adolescent cohort, again using an ANOVA. Cortical integration of the STN differed significantly between patients with chronic pain and healthy controls (*F(49,1)* = 7.242, p <0.01). Exploratory, we also calculated the same tests for the remaining basal ganglia, thalamus, and caudate regions. The thalamus lateral and middle, the caudate anterior and superior, as well as the right hemisphere’s substantia nigra, globus pallidus interna and striatum showed significantly (p_adj_ ≤0.05) altered cortical integration (Figure 3A).

## Discussion

Painful symptoms during adolescence have been linked to an increased susceptibility to chronic pain in adulthood,^19^ and chronic pain in adulthood has been associated with alterations in subcortical-cortical brain circuitry.^1^^,^^2^^,^^3^ A potential link between these observations may be seen in a pain-associated ontogenic organization of subcortical-cortical circuitry in adolescence, which might overlap with brain circuits implicated in pain chronicity. To this end, our study sought to investigate the effect of ontogenic brain organization on painful symptoms in late adolescence, specifically focusing on subcortical-cortical circuitry and to investigate whether this adolescents’ pain-related ontogeny shares a neural basis with functional alterations in brain organization in adult chronic pain compared to healthy controls.

We provide evidence that ontogenic basothalamo-cortical organization is associated with painful symptoms in late adolescence and that the pattern of brain connectivities associated with painful symptoms overlaps with basothalamo-cortical connectivity alterations in adult chronic pain. The findings suggest ontogenic brain changes as a potential marker of the existence of pain symptoms early in life, which could represent a risk marker for and/or reflect adaption processes related to chronic pain later in life, during adulthood. A clear causal interference between the two brain-pain associations can, however, not be made. This should be further investigated in future studies.

The examination on pain representation in adolescents has yet primarily focused on cortical areas and single time points.^16^^,^^17^^,^^18^ However, previously we found a predictive role of periaqueductal gray and striatum responses to reward processing in pain symptoms during adolescence.^15^ Notably, the structures’ functional changes we found being associated with pain in this study were also subcortical basal ganglia areas. This further indicates a particular role of subcortical and basal ganglia structures in the development of pain-related symptoms during adolescence.

The ontogenic connectivity changes of the STN were exclusively negatively associated with pain symptoms in late adolescence. The STN is related to pain processing through thalamocortical pathways and highly connected to cortical areas,^20^^,^^21^^,^^22^ thereby transferring cortical modulation on thalamocortical pathways, that relay nociceptive spinal cord signals to the cortex.^21^^,^^23^ Accordingly, the observed negative associations between the change in the cortical integration of the STN and painful symptoms may represent increased cortically controlled the inhibition of the thalamocortical pathway via the indirect or hyperdirect pathways of the STN.

This is also important with respect to the findings in our chronic pain sample. The increased cortical connectivity of the STN together with thalamocortical hyperconnectivity, we observed in chronic pain, might represent an increased effort of the indirect or hyperdirect pathways of the STN indicated by the increased cortico-subthalamic connectivity. In this case within the group of patients with chronic pain a higher ratio of cortico-subthalamic to thalamo-cortical connectivity should be associated with lower individual sensory pain perception. We tested this at the post-hoc level, and indeed found a negative association between the individual attention to sensory changes in pain (*r(27)* = -0.40, p ≤ 0.04) and the ratio of subthalamic and thalamic cortical integration. The observed hyperconnection between the caudate and right hemispheric striatum, globus pallidus and substantia nigra, all belonging to the baso-thalamic pathways, in adult chronic pain additionally supports the role of these pathways for painful symptoms in adolescence and chronic pain in adulthood.

In addition to the basal ganglia and thalamic pathways, in the adolescent cohort we also found the left RN as hub for cortical connectivity positively associated with painful symptoms. The RN has been studied mainly in terms of motor functions and other functions are suspected although largely unknown.^24^ For pain the observed positive associations of the RN may represent increased descending cortical projections, that inhibit the antinociceptive effect of the RN mediated by connections to the periaqueductal gray.^24^ The role of the RN in pain processing should be the subject of future research.

With respect to the RN and the STN and their implication in motor pathways,^25^ it is important to note that the painful symptom score of the Children Somatisation Inventory (CSI-24), used in the adolescent sample, also includes items on feelings of weakness and fatigue.^26^^,^^27^ In chronic pain, the interplay between pain and motor weakness is known to be exaggerated and leads to general symptoms of tiredness and fatigue.^28^ The significant neural representation of symptoms of both pain and motor-related impairments in an epidemiological sample with low symptom severity underlines the close relationship between motor and pain systems, also outside of pathological pain conditions.

In previous research we showed that activity in subcortical basal ganglia structures, the striatum and periaqueductal gray, predict painful symptoms in early adolescence.^15^ Our results in this article show that painful symptoms in late adolescence are associated with connectivities of particularly basothalamo-cortical circuitry and we did not find significant association in the amygdala-nucleus accumbens-cortex complex, which has been shown to be predictive of pain persistence in the transfer of subacute to chronic pain.^7^^,^^8^^,^^9^ However the same chronic pain and healthy control cohort that we included in this analysis did also not show altered connectivity in this complex, either during a reward task or at resting state.^8^ Taken together this suggests that the amygdala-nucleus accumbens-cortex complex comes into play specifically during the transfer of subacute to chronic pain probably due to its prominent role in operant learning and is not engaged in late adolescent regular pain symptomatology nor in chronic pain.

Our analysis on adolescence as sensitive period for pain aimed to identify changes in brain connectivity associated with painful symptoms. This approach focuses on the effect of ontogenic organization but does not assess time point-specific neural correlates as previous research primarily did.^16^^,^^18^ For compatibility with these studies as supplemental analysis we also associated rsFCs at each time point with the concurrent painful symptoms score using an ordinary-least-square regression with sex as additional variable as confounder. We found only marginal brain connectivity correlates of painful symptoms at TP1, while at TP2 the brain circuits of the brain connectivities whose change correlated with pain symptoms also widely correlated with the painful symptoms (Figure S2 and Table S1). Furthermore, the average association (β) significantly increased from TP1 to TP2 (Table S2). This again emphasizes late adolescence as be a sensitive period for pain-related neural representations.

While we did not aim to assess, whether the pain-related ontogenic brain organization can also be used to classify individuals with high painful symptoms scores, determining the classification information might still be interesting, given the literature on the longitudinal changes from (sub)acute to chronic (or persistent) pain. We therefore calculated receiver operation characteristics according to previous studies (e.g.,^8^) and showed that the classification of the change in cortical STN integration was higher than chance level. Since, our sample is an epidemiological sample with comparably low symptom severity and variability, findings are not directly comparable to measures known from predicting persistence of subacute pain through prefrontal-limbic interactions.^7^^,^^8^ However, the provide some first evidence that such pain chronicity classification might already be useful and informative at these earlier stages. Future research should address this aspect in more detail.

### Limitations of the study

It is important to acknowledge that, while we can argue in terms of a common neural basis of regular pain symptoms in adolescence and chronic pain, we cannot directly infer the causality of altered basothalamo-cortical organisation in chronic pain from adolescence to adulthood. This was also not the aim of the present study. Our results provide evidence of a common neural basis during adolescence, a period, in which pain problems just start to develop, and in adults, where this pain symptoms development has proceeded and who suffer from chronic pain. These brain alterations might thus serve as an indicator of a high risk for the development of chronic pain. Since the existence of pain also induces in several individual coping behaviors, it might also be the case that this identified brain patterns reflects an adaptation process to pain. To further process in this, longitudinal studies are needed, also from the sensitive period of (sub)acute to chronic pain. Moreover, in the present study, we exclusively used methods of resting-state brain imaging, and thus do not have information on parallel behavioral processes. However, we aimed to analyze representations of painful symptoms in trait-like brain organization for which resting-state imaging when combined with connectomics is the most informative tool in functional neuroimaging. Nevertheless, to define the role of the STN and RN and their connectivities, future studies should address behavioral performances together with brain imaging along activity and particularly dynamic connectivity approaches in event-related paradigms. Furthermore, we need to consider that, in our adolescent population, the mean score of painful symptoms decreases (Table S3). Although we included the individual values of decreasing and increasing symptom scores in our analyses from our data it cannot be directly inferred whether the brain representations are pain-associated or coping-related. In this regard, it should also be noted that the CSI used here has so far only been validated in younger samples of up to 17 years old. Since there is no conflict with respect to the validity of the item content and age, and based on the TP1 data quality controls, CSI has been used for all the assessment time point in IMAGEN to keep the longitudinal chain and study pipeline. Nevertheless, we cannot completely rule out a potential bias. However, by demonstrating that the same regions are altered in chronic pain we can emphasize the role of these in chronic pain either way and by referring to experimentally validated models of thalamic and basal ganglia pathways we suggest a mechanistic explanation to our results.

In conclusion, our results indicate a shared basis in the basothalamo-cortical circuitries between painful symptoms in adolescent and adult pain chronicity suggesting complex brain-pain trajectories. This suggests late adolescence as sensitive periods for the emergence of this (chronic) pain associated neural circuitry through the ontogenic organization of pain-associated thalamic and basal ganglia pathways. Future research should investigate these as potential risk factors for chronic pain in adulthood.

## Consortia

IMAGEN Consortium: Tobias Banaschewski, Gareth J. Barker, Arun L. W. Bokde, Herta Flor, Antoine Grigis, Hugh Garavan, Penny Gowland, Andreas Heinz, Rüdiger Brühl, Jean-Luc Martinot, Eric Artiges, Frauke Nees, Dimitri Papadopoulos Orfanos, Herve Lemaitre, Tomáš Paus, Luise Poustka, Lauren Robinson, Sarah Hohmann, Juliane H. Fröhner, Michael N. Smolka, Henrik Walter, Robert Whelan, Jeanne Winterer, Edward D. Barker & Gunter Schumann.

## STAR★Methods

### Key resources table

**Table:** 

REAGENT or RESOURCE	SOURCE	IDENTIFIER
**Software and algorithms**		

fMRIprep 20.2.3 / 21.0.4	Poldrack Lab	RRID:SCR_016216
statsmodels	Python Package	RRID:SCR_016074
NiLearn	Python Package	RRID:SCR_001362
Python3	Python	RRID:SCR_008394

**Deposited data**		
Original analysis code	this paper	https://doi.org/10.5281/zenodo.6554137

### Resource availability

#### Lead contact

Requests for resources should be directed to and will be fulfilled by the lead contact, Frauke Nees (nees@med-psych.uni-kiel.de).

#### Materials availability

This study did not generate new unique reagents.

#### Data and code availability

• All data needed to evaluate the conclusions in the paper are present in the paper and/or the supplemental information. The data that support the findings of this study are available from the IMAGEN-Consortium, but restrictions apply to the availability of these data, which were used under license for the current study, and so are not publicly available. Data are, however, available from the authors upon request and with permission of the IMAGEN-Consortium. The data used for the adult cohort are available upon request from the lead contact.
• All original code has been deposited at Zenodo and is publicly available as of the date of publication via Zenodo: https://doi.org/10.5281/zenodo.6554137. All analyses were computed using Python 3.^29^ For the main analysis, the packages nilearn^30^ and statsmodels^31^ were used.
• Any additional information required to reanalyze the data reported in this paper is available from the lead contact upon request.

### Experimental model and study participant details

#### Participants

In the present study, data from two independent cohorts were used.

##### Adolescent cohort

We used data from a large longitudinal sample from the EU-IMAGEN project^32^ which was recruited from the public through school visits, flyers, and registration offices in 8 centres in Germany, the United Kingdom, Ireland, and France. For the present paper, we examined a subgroup of 690 volunteers (399 females) at the age of 18-20 (TP1) and 21-24 years (TP2), for whom variables of interest were consistently present. Of all participants 599 had two Caucasian parents, 62 had one non-Caucasian parent and 26 had two non-Caucasian parents. For 3 participants no ancestry data was present. Exclusion criteria were as follows: any mental disorder as defined by the Development and Well-Being Assessment (DAWBA^33^) contraindications for magnetic resonance imaging (MRI) examinations, serious medical conditions, pregnancy, and previous head trauma with unconsciousness. The study was approved by the local ethics committees and adhered to the Declaration of Helsinki. After a complete description of the study, written informed consent was obtained from the participants.

##### Adult cohort

Resting-state data from participants for the adult cohort has already been used in earlier publication.^8^ In this analysis we included the same 29 patients with chronic back pain (CBP) and 29 age and sex-matched healthy controls (HC). Ancestry, race, or ethnicity were not assessed. For the CBP group we only included patients with a current back pain episode of more than 100 days. Patients were assessed for general eligibility via self-report using a screening intake form, which covered co-morbid health and psychological conditions, MRI safety, concomitant medication dosages and indications, current and previous illicit drug/alcohol use, and pain levels. All participants passed the MRI safety screening requirements at each scanning visit. Informed consent was obtained from all participants on their first visit. Participants were compensated with €10/hour. All procedures were approved by the Ethics Committee of the Medical Faculty Mannheim of Heidelberg University and complied with the Declaration of Helsinki in its most recent form. Resting state scans from two patients with CBP and four HC were not available, which were therefore excluded from analysis. To assess comorbid mental disorders, all participants were interviewed by a psychologist using the German version of the Structured Clinical Interviews (SCID I)^34^ for the Diagnostic and Statistical Manual of Mental Disorders (DSM IV),^35^ see Table S4 for all current or past disorders and for details on medication see Table S5.

### Method details

#### Assessments of painful symptoms and participant characteristics

At TP1 and TP2, participants of the Adolescent cohort completed the Children Somatization Inventory (CSI)^36^ to assess the perceived severity of symptoms, including items for pain. For TP1, we used the CSI-35, which consists of 35 items from the symptom criteria for somatization disorder as defined by DSM-III-R^37^ and somatization factor of the Hopkins Symptom Checklist (HSCL)^38^ as well as additional symptoms that are common in functional gastrointestinal disorders. The response format was a 5-point scale ranging from “not at all” (0) to “a whole lot” (4) and the standard period for symptom reports is 2 weeks. At TP2, the shortened form of the CSI consisting of 24 items^39^ was applied. For indicators of pain, we calculated a painful symptom score following a multiply reproduced factor structure for the CSI-24, which integrates items on pain complaints, including complaints on motor weakness symptoms^26^^,^^27^ for each time point and is also applicable to the CSI-35 version. The CSI was implemented in Psytools software (Delosis Ltd, London, United Kingdom), an online battery that participants completed at home.

Within the group of chronic pain patients in the adult cohort we queried the Individual attention to sensory changes in pain using the German version of the pain vigilance and awareness questionnaire^40^ with the subscale attention to changes in pain. Furthermore the German version of the Hospital Anxiety and Depression Scale^41^ was conducted to describe individual characteristics and mental well-being. Participant characteristics for both cohorts are stated in Tables S3 and S6.

#### MRI-acquisition

##### Adolescent-cohort

Scanning for the IMAGEN-cohort was performed with a 3T whole-body MRI system made by several manufacturers (Siemens, Philips, General Electric, and Bruker) at the 8 IMAGEN assessment sites (Paris, London, Dublin, Nottingham, Dresden, Berlin, Mannheim, and Hamburg). A key challenge for the ability to pool data acquired on MR scanners of different manufacturers relates to their variation in availability and implementation of particular image-acquisition techniques. To address this problem, for each technique, a set of parameters compatible with all scanners, particularly those directly affecting image contrast or signal-to-noise, was devised and held constant across sites. Where manufacturer-specific choices had to be made (for example, the design of head coil), the best manufacturer-specific option was used at all sites with the same scanner type (for further details, please refer to^32^).

For the resting-state assessments, 40 slices in descending order (2.4 mm, 1 mm gap) were acquired using a gradient-echo echo-planar (EPI) LA∗-weighted sequence (TR=2200 milliseconds, TE=30 milliseconds, in-plane resolution of 64 x 64 pixels). We used a plane of acquisition tilted to the anterior-posterior commissure line (rostral-caudal). Moreover, a 3D magnetization prepared gradient-echo sequence based on the Alzheimer’s Disease Neuroimaging Initiative (ADNI) protocol (http://adni.loni.usc.edu/methods/documents/mri-protocols/) over the whole brain was performed for anatomical reference.

##### Adult cohort

Magnetic resonance imaging was performed on a 3 Tesla Tim TRIO whole body scanner (SIEMENS Healthineers, Erlangen, Germany), equipped with a 12-channel head coil. Shimming of the scanner was done to account for maximum magnetic field homogeneity and a standard gradient field map was recorded at the beginning of each measurement. For the resting-state functional protocol, 36 contiguous axial slices (slice thickness: 3 mm, slice, in-plain voxel size: 2.3 × 2.3 mm, no gap) were acquired using a T2∗-weighted gradient-echo echo-planar imaging (EPI) sequence with GRAPPA technique (acceleration factor 2, repetition time (TR) = 2100 ms, echo time (TE) = 23 ms, matrix size = 96 × 96, field of view (FoV) = 220 × 220 mm2, flip angle (α) = 90°, bandwidth (BW) = 1370 Hz/px). Two-hundred-and ten volumes were acquired in a total of 7 min and 21 s. Subjects were asked to lay still with their eyes closed, remain awake and try not to think about anything specific. For structural reference, we used a T1-weighted magnetization prepared rapid gradient echo (MPRAGE) sequence (TR = 2300 ms, TE = 2.98 ms, matrix size = 240 × 256, field of view (FoV) = 240 × 256 mm2, flip angle (α) = 9°, bandwidth (BW) = 240 Hz/px) recording with 192 sagittal slices.

### Quantification and statistical analysis

#### Functional magnetic resonance imaging preprocessing and denoising

In the adolescent cohort we preprocessed the MRI data using fMRIPrep 20.2.3^42^ with non-aggressive ICA-Aroma strategy. We ran fMRIPrep individually for TP1 and TP2 to optimally account for possible anatomical changes between the measurements. Due to partially incomplete data no slice-time-correction and susceptibility distortion correction was applied. In the adult cohort fMRIPrep 21.0.4 was used and slice time correction was applied. A complete fMRIPrep-boilerplate follows (20.2.3).

Results included in this manuscript come from preprocessing performed using *fMRIPrep* 20.2.3 and 21.0.4^42^^,^^43^ (RRID:SCR_016216), which is based on *Nipype* 1.6.1^44^^,^^45^ (RRID:SCR_002502).

#### Anatomical data preprocessing

A total of 1 T1-weighted (T1w) images were found within the input BIDS dataset. The T1-weighted (T1w) image was corrected for intensity non-uniformity (INU) with N4BiasFieldCorrection,^46^ distributed with ANTs 2.3.3^47^ (RRID:SCR_004757), and used as T1w-reference throughout the workflow. The T1w-reference was then skull-stripped with a *Nipype* implementation of the antsBrainExtraction.sh workflow (from ANTs), using OASIS30ANTs as target template. Brain tissue segmentation of cerebrospinal fluid (CSF), white-matter (WM) and gray-matter (GM) was performed on the brain-extracted T1w using fast^48^ (FSL 5.0.9, RRID:SCR_002823). Brain surfaces were reconstructed using recon-all^49^ (FreeSurfer 6.0.1, RRID:SCR_001847), and the brain mask estimated previously was refined with a custom variation of the method to reconcile ANTs-derived and FreeSurfer-derived segmentations of the cortical gray-matter of Mindboggle^50^ (RRID:SCR_002438). Volume-based spatial normalization to two standard spaces (MNI152NLin2009cAsym, MNI152NLin6Asym) was performed through nonlinear registration with antsRegistration (ANTs 2.3.3), using brain-extracted versions of both T1w reference and the T1w template. The following templates were selected for spatial normalization: *ICBM 152 Nonlinear Asymmetrical template version 2009c* [^51^, RRID:SCR_008796; TemplateFlow ID: MNI152NLin2009cAsym], *FSL’s MNI ICBM 152 non-linear 6th Generation Asymmetric Average Brain Stereotaxic Registration Model* [^52^, RRID:SCR_002823; TemplateFlow ID: MNI152NLin6Asym].

#### Functional data preprocessing

For each of the 1 BOLD runs found per subject (across all tasks and sessions), the following preprocessing was performed. First, a reference volume and its skull-stripped version were generated using a custom methodology of *fMRIPrep*. Susceptibility distortion correction (SDC) was omitted. The BOLD reference was then co-registered to the T1w reference using bbregister (FreeSurfer) which implements boundary-based registration.^53^ Co-registration was configured with six degrees of freedom. Head-motion parameters with respect to the BOLD reference (transformation matrices, and six corresponding rotation and translation parameters) are estimated before any spatiotemporal filtering using mcflirt (FSL 5.0.9,^54^). The BOLD time-series (including slice-timing correction when applied) were resampled onto their original, native space by applying the transforms to correct for head-motion. These resampled BOLD time-series will be referred to as *preprocessed BOLD in original space*, or just *preprocessed BOLD*. The BOLD time-series were resampled into standard space, generating a *preprocessed BOLD run in MNI152NLin2009cAsym space*. First, a reference volume and its skull-stripped version were generated using a custom methodology of *fMRIPrep*. Automatic removal of motion artifacts using independent component analysis (ICA-AROMA,^55^) was performed on the *preprocessed BOLD on MNI space* time-series after removal of non-steady state volumes and spatial smoothing with an isotropic, Gaussian kernel of 6mm FWHM (full-width half-maximum). Corresponding “non-aggresively” denoised runs were produced after such smoothing. Additionally, the “aggressive” noise-regressors were collected and placed in the corresponding confounds file. Several confounding time-series were calculated based on the *preprocessed BOLD*: framewise displacement (FD), DVARS and three region-wise global signals. FD was computed using two formulations following Power (absolute sum of relative motions,^56^) and Jenkinson (relative root mean square displacement between affines,^54^). FD and DVARS are calculated for each functional run, both using their implementations in *Nipype* (following the definitions by^56^). The three global signals are extracted within the CSF, the WM, and the whole-brain masks. Additionally, a set of physiological regressors were extracted to allow for component-based noise correction (*CompCor*,^57^). Principal components are estimated after high-pass filtering the *preprocessed BOLD* time-series (using a discrete cosine filter with 128s cut-off) for the two *CompCor* variants: temporal (tCompCor) and anatomical (aCompCor). tCompCor components are then calculated from the top 2% variable voxels within the brain mask. For aCompCor, three probabilistic masks (CSF, WM and combined CSF+WM) are generated in anatomical space. The implementation differs from that of Behzadi et al. in that instead of eroding the masks by 2 pixels on BOLD space, the aCompCor masks are subtracted a mask of pixels that likely contain a volume fraction of GM. This mask is obtained by dilating a GM mask extracted from the FreeSurfer’s *aseg* segmentation, and it ensures components are not extracted from voxels containing a minimal fraction of GM. Finally, these masks are resampled into BOLD space and binarized by thresholding at 0.99 (as in the original implementation). Components are also calculated separately within the WM and CSF masks. For each CompCor decomposition, the *k* components with the largest singular values are retained, such that the retained components’ time series are sufficient to explain 50 percent of variance across the nuisance mask (CSF, WM, combined, or temporal). The remaining components are dropped from consideration. The head-motion estimates calculated in the correction step were also placed within the corresponding confounds file. The confound time series derived from head motion estimates and global signals were expanded with the inclusion of temporal derivatives and quadratic terms for each.^58^ Frames that exceeded a threshold of 0.5 mm FD or 1.5 standardised DVARS were annotated as motion outliers. All resamplings can be performed with *a single interpolation step* by composing all the pertinent transformations (i.e. head-motion transform matrices, susceptibility distortion correction when available, and co-registrations to anatomical and output spaces). Gridded (volumetric) resamplings were performed using antsApplyTransforms (ANTs), configured with Lanczos interpolation to minimize the smoothing effects of other kernels.^59^ Non-gridded (surface) resamplings were performed using mri_vol2surf (FreeSurfer).

Many internal operations of *fMRIPrep* use *Nilearn* 0.6.2 (^30^, RRID:SCR_001362), mostly within the functional processing workflow. For more details of the pipeline, see *the section corresponding to workflows in fMRIPrep’s documentation*.

The above boilerplate text was automatically generated by fMRIPrep with the express intention that users should copy and paste this text into their manuscripts unchanged. It is released under the creative commons (https://creativecommons.org/publicdomain/zero/1.0/).

We then denoised the preprocessed images using the ICA-Aroma-8Phys strategy, which in a data-driven way robustly identifies various noise sources^60^ and thus is particularly suitable to denoise fMRI data integrated from varying equipment. Furthermore, images were bandpass filtered (0.1Hz, 0.01Hz) and spatially smoothed (5mm).

#### Resting-state functional connectivity and resting-state functional connectivity change

Individual unstandardized whole-brain functional connectomes were computed using pair-wise Pearson correlation. Two parcellations were utilized to separate the brain into regions of interest (ROI) for constructing the individual connectomes. The DiFuMo-256-parcellation was implemented to reduce whole-brain dimensionality by separating the cortex into functionally representative cortical areas.^61^ A Basal Ganglia parcellation^62^ was implemented to represent relevant areas of the nociceptive and pain-modulatory structures. For the IMAGEN-cohort we used the parcellation for young persons, in the clinical cohort the one for middle aged persons to account for anatomical changes.^62^ Change in rsFC during late adolescence in the IMAGEN-cohort was operationalized as the arithmetic difference of the rsFCs between TP2 and TP1 (ΔrsFC).

#### Statistical analysis

##### Association of functional connectivity change and painful symptoms in late adolescence

We aimed to correlate painful symptom scores at TP2 with the ontogenic functional brain organization during late adolescence, operationalized as the difference of rsFCs between TP1 and TP2, with. We therefore used every ΔrsFC as a regressor for painful symptoms at TP2, while explicitly controlling for rsFC at TP1 and sex as additional factors in an ordinary least-square regression (Equation 1)

(Equation 1) $$Painful\;symptoms\left(T_{\text{TP}2}\right)\;∼\Delta \text{rsFC}\left(T_{\text{TP}2},T_{\text{TP}1}\right)+\text{rsFC}\left(T_{\text{TP}1}\right)+\text{sex}+C$$

##### Association of cortical integration change of the STN and painful symptoms in late adolescence

Due to the many negative associations of the rsFC of the STN and various cortical areas with painful symptom scores (see results), we also calculated the association of the ontogenic cortical integration of the STN during late adolescence with painful symptoms. Ontogenic cortical integration was calculated as the mean ΔrsFC to the ROIs defined by the DiFuMo-parcellation. We then correlated the painful symptom scores at TP2 with the change of mean ΔrsFC and the, while controlling for mean rsFC at TP1 (Equation 2) using an ordinary least-square regression.

(Equation 2) $$Painful\;symptoms\left(T_{\text{TP}2}\right)\;∼\;mean\left(\Delta {\text{rsFC}}_{STN,i}\right)+{\text{mean}(\text{rsFC}}_{STN,i}\left(T_{\text{TP}1}\right))+\text{sex}+C$$

To also test, to which extant the change of cortical integration of the STN distinguishes between adolescents, which are affected by comparably higher amounts of painful symptoms and those which are not receiver operating characteristics and area under the curve were calculated for percentiles from median to maximum in 5% steps.

##### Differences of brain connectivity in adult chronic pain compared to healthy controls

RsFC and cortical integration of single ROIs in the adult cohort were operationalized like in the adolescent sample. We then tested for differences between groups. To control for sex, we used ANOVAs with chronic pain and sex as independent variables for the connectivity parameters. Only the results of interest concerning the group factor are reported.

##### Statistical significance and multiple-comparison correction

All tests were performed with a significance level of α=0.05, two-tailed. In the connectome-wide association tests we controlled for multiple comparison using a ROI-level false discovery rate correction based on the Benjamini-Hochberg-procedure. This correction was applied independently to each row of the connectivity matrix corresponding to a specific ROI. The use of this relatively liberal correction approach aims to enhance sensitivity for small effects, which are of interest in non-pathological population studies and may be prone to conservative testing methods. Furthermore, unlike network-based correction approaches, which prioritize large and strongly associated clusters of ROIs, the ROI-wise correction method is also sensitive to effects involving ROIs with fewer associated counterparts, as expected in subcortical pain modulatory structures, particularly the ROIs of the basal ganglia.
