# Supplementary material for: Adolescents’ pain-related ontogeny shares a neural basis with adults’ chronic pain in basothalamo-cortical organization
Source: iScience. 2024 Jan 17;27(2):108954. doi: 10.1016/j.isci.2024.108954 (PMC10845062; doi:10.1016/j.isci.2024.108954)
Supplement: Document S2. Figures S1 and S2 and Tables S1–S6 [file mmc1.pdf]

iScience, Volume *27*

## Supplemental information

# Adolescents' pain-related ontogeny shares a neural basis with adults' chronic pain in basothalamo-cortical organization

**Nils Jannik Heukamp, Tobias Banaschewski, Arun L.W. Bokde, Sylvane Desrivières, Antoine Grigis, Hugh Garavan, Penny Gowland, Andreas Heinz, Mina Kandić, Rüdiger Brühl, Jean-Luc Martinot, Marie-Laure Paillère Martinot, Eric Artiges, Dimitri Papadopoulos Orfanos, Herve Lemaitre, Martin Löffler, Luise Poustka, Sarah Hohmann, Sabina Millenet, Juliane H. Fröhner, Michael N. Smolka, Katrin Usai, Nilakshi Vaidya, Henrik Walter, Robert Whelan, Gunter Schumann, Herta Flor, Frauke Nees, and IMAGEN Consortium**

**This PDF file includes:**

Table S1. Number of significantly associated rsFC and ∆rsFCs for single ROIs. Related to Figure 1.

Table S2. Mean association (β) of rsFCs with painful symptoms at TP1 and TP2 as well as their difference. Related to Figure 1 and 2.

Table S3. Participant characteristics of the adolescent cohort at TP1 and TP2. Related to Star Methods.

Table S4. Current or past mental disorders in the adult cohort. Related to Star Methods.

Table S5. Medication in the adult cohort. Related to Star Methods.

Table S6. Participant characteristics of the adult cohort. Related to Star Methods.

Figure S1. Receiver operating characteristics and area under the curve for multiple percentile thresholds. Related to Figure 1 and 2.

Figure S2. Brain Connectivity Correlates of Painful Symptoms at TP1 and TP2. Related to Figure 1 and 2.

**Table S1. Number of significantly associated rsFC and ∆rsFC for single rois for supplemental timepoint-specific analysis and main prediction model. Related to Figure 1.**

| **ROI[a]** | | Timepoint-specific models | | Prediction model | |
|---|---|---|---|---|---|
| anatomical classification | DiFuMo-ROI-number | number of significantly associated rsFC at TP1 (βα≤.05) | number of significantly associated rsFC at TP2 (βα≤.05) | number of significantly associated rsFC at TP1 (βα≤.05) | number of significantly associated ∆rsFC (βα≤.05) |
| subthalamic nucleus rh | - | | 107 | | 97 |
| red nucleus lh | - | | 67 | | 84 |
| thalamus middle | 41 | | 11 | | 42 |
| caudate inferior | 37 | | 40 | | 38 |
| middle frontal gyrus | 136 | | 40 | | 32 |
| thalamus inferior | 206 | | 17 | | 20 |
| hippocampus anterior | 168 | | 20 | | 20 |
| frontomarginal gyrus | 92 | | 15 | | 13 |
| ventromedial prefrontal cortex | | | 9 | | 6 |
| middle temporal gyrus anterior lh | 134 | | 5 | | 9 |
| planum temporale rh | 204 | | 4 | | 5 |
| precentral sulcus superior lh | 221 | | 5 | | 6 |
| occipitotemporal gyrus anterior | 237 | | 6 | | 6 |
| anterior horizontal ramus lateral fissure rh | 242 | | 4 | | 4 |
| collateral sulcus anterior | 10 | | | | 4 |

| | | | | | |
|---|---|---|---|---|---|
| pars opercularis pars triangularis lh | 16 | | | | 4 |
| suborbital cortex | 56 | | | | 4 |
| hippocampus posterior | 95 | | | | 4 |
| temporal pole | 170 | | | | 4 |
| precentral sulcus inferior lh | 171 | | | | 7 |
| superior temporal sulcus rh | 186 | | | | 4 |
| insula posterior inferior | 190 | | | | 5 |
| central opercular cortex | 191 | | | | 4 |
| cingulate sulcus posterior | 201 | | | | 4 |
| middle frontal gyrus rh | 245 | | | | 4 |
| heschl’s gyrus | 86 | | | | 6 |
| genu of callosal body | 60 | | | 14 | |
| anterior comissure | 66 | 3 | | | |
| total | | 3 | 308 | 17 | 346 |

[a]*ROIs with at least 3 significant associations in one of the analyses are listed.*

**Table S2. Mean association (β) of rsFCs with painful symptoms at TP1 and TP2 as well as their difference. Related to Figure 1 and 2.**

| | n | mean | std | *t*(36314) | p | Cohen's d |
|---|---|---|---|---|---|---|
| TP1 | 36315 | -.004 | 0.035 | -21.126 | <.001 | -.110 |
| TP2 | 36315 | .028 | 0.043 | 123.972 | <.001 | .651 |
| TP2 - TP1 | 36315 | .032 | .048 | 126.417 | <.001 | .794 |

**Table S3. Participant characteristics of the adolescent cohort at TP1 and TP2. Related to Star Methods.**

| | TP1 | TP2 | Difference |
|---|---|---|---|
| Gender | 399/291 | | - |
| Age | 18.4 (.7) | 22.0 (.68) | 3.55 (0.76) |
| Painful symptom score[a] | 7.00% (8.50%) | 5.30% (8.40%) | -1.70% (8.40%) |

[a] *Painful symptom score as the percentage of the maximal possible score.*

**Table S4. Current or past mental disorders in the adult cohort. Related to Star Methods.** Diagnoses according to the Diagnostic and Statistical Manual of Mental Disorders IV (DSM IV)

| | Code | Diagnosis | Remitted | Acute |
|---|---|---|---|---|
| Healthy Controls | 296.26 | Major depressive disorder, single episode | 1 | |
| Chronic Back Pain | 296.26 | Major depressive disorder, single episode | 3 | 2 |
| | 296.33/296.36 | Major depressive disorder, recurrent | 3 | 2 |
| | 300.01 | Panic disorder, without agoraphobia | | |
| | 300.22 | Agoraphobia without history of panic disorder | 1 | |
| | 303.90 | Dependence: Alcohol | 1 | |
| | 304.10 | Dependence: Sedative-, hypnotic-, or anxiolytic-related | 1 | |
| | 305.xx | Abuse: Cannabis, Cocaine, Hallucinogen, Amphetamine | 1 | |
| | 307.10 | Anorexia Nervosa | | 1 |
| | 307.51 | Bulimia Nervosa | 1 | |

**Table S5. Medication in the adult cohort. Related to Star Methods.**

| | Medication | N(CBP) | N(HC) |
|---|---|---|---|
| Current Medication | NSAID | 3 | |
| | antihistamines | 1 | |
| | angiotensin receptor blockers | 1 | |
| | Benzodiazepines | 1 | |
| Past Medication | NSAIDs | 14 | 1 |
| | Opiates | 2 | |
| | ACE inhibitors | 1 | |
| | Benzodiazepines | 1 | |

**Table S6. Participant characteristics of the adult clinical cohort. Related to Star Methods.**

| | CBP | HC | Missing |
|---|---|---|---|
| N | 29 | 29 | NA |
| Age | 38.7 (15.8) | 35.7 (14.7) | 0/0 |
| Gender (m/f) | 16/13 | 17/12 | 0/0 |
| Anxiety (HADS) | 7.32 (4.50) | 3.54 (2.39) | 4/3 |
| Depression (HADS) | 5.59 (4.08) | 2.96 (4.24) | 4/3 |

*All values show the mean and standard deviation.*

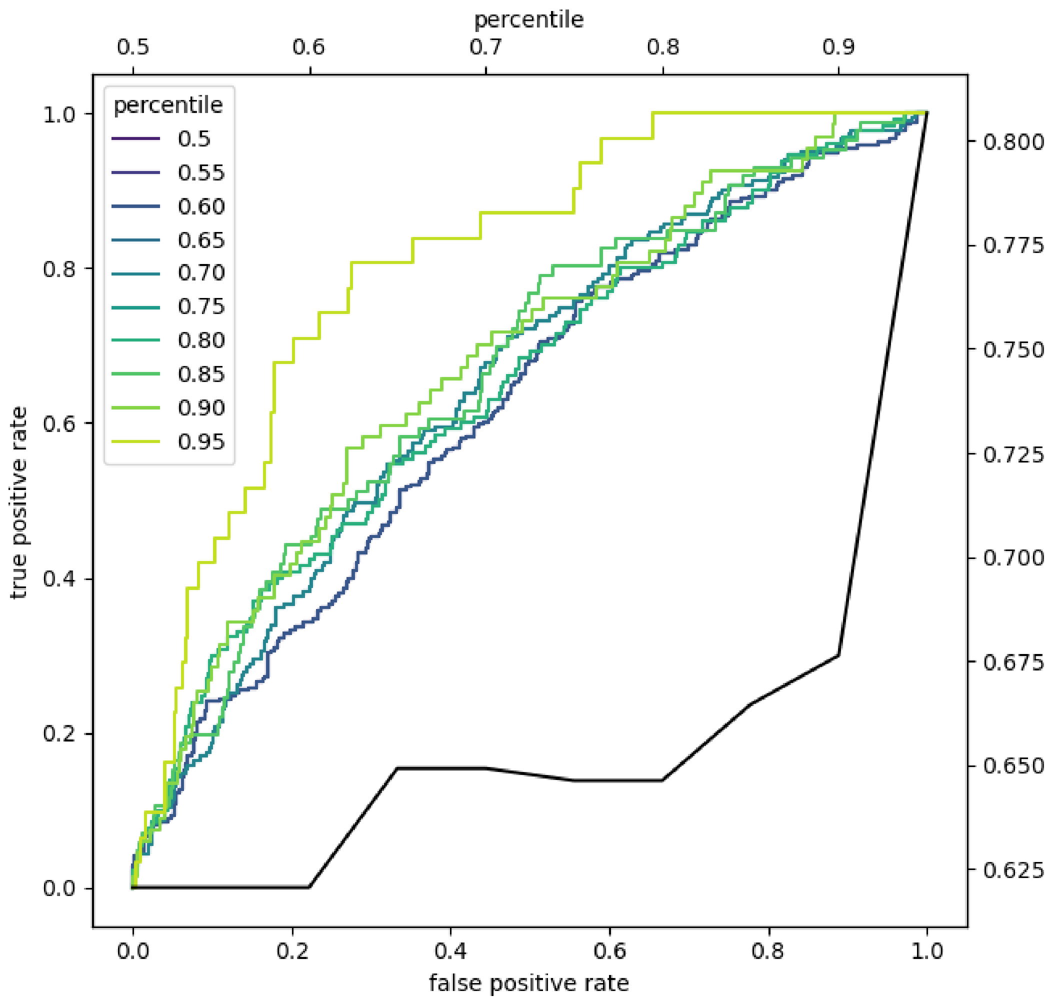


**Figure S1. Receiver operating characteristics and area under the curve for multiple percentile thresholds.** Related to Figure 1 and 2. True positive rate (y-axis, left) dependent on false positive rate (x-axis, bottom) as a function of different percentile thresholds (green lines) as well as the area under the curve (y-axis, left) as a function of the percentile threshold (x-axis, top) depicted as the black line. ROC: receiver operating characteristics; AUC: area under the curve.

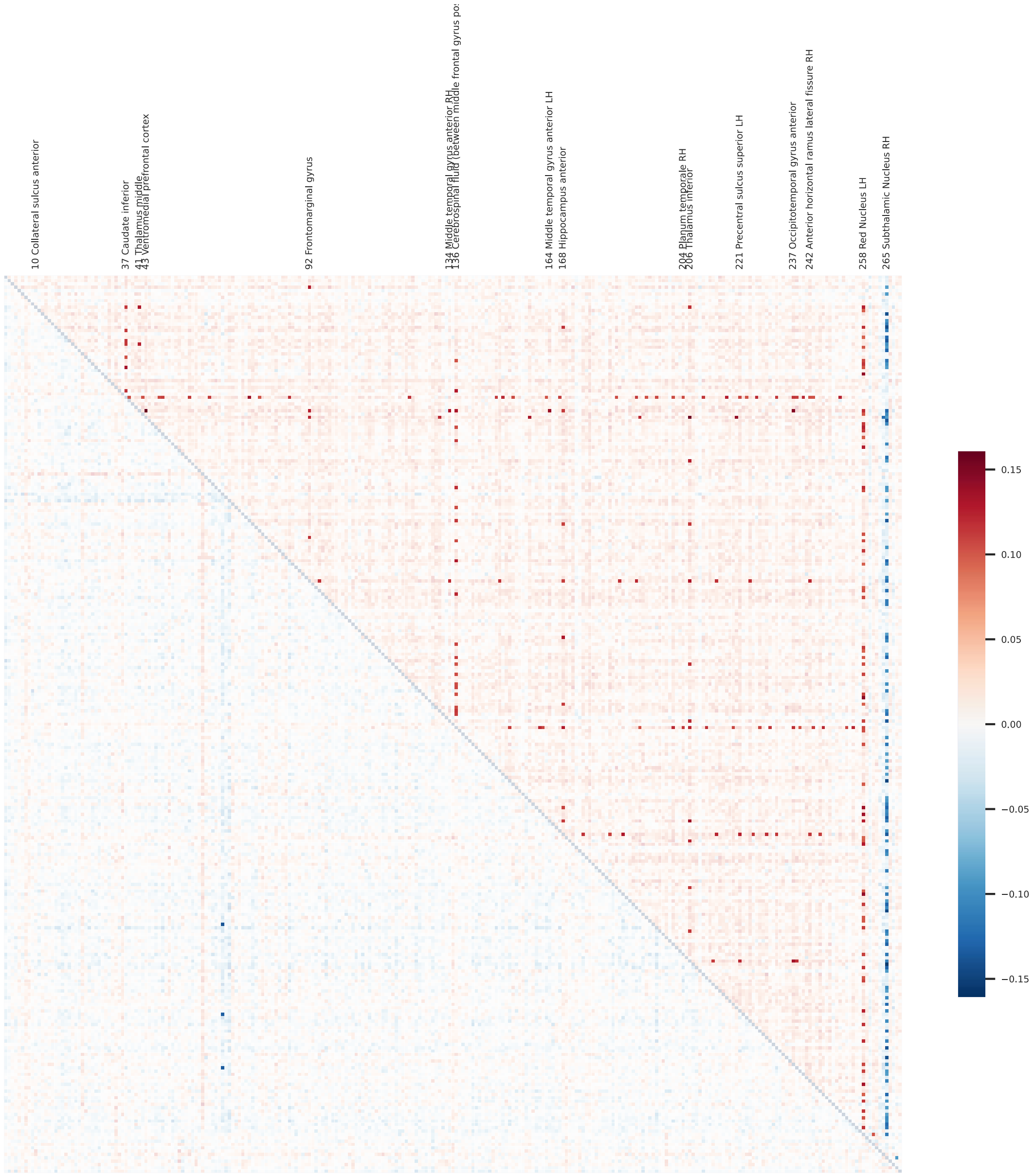


**Figure S2. Ontogenic Basothalamo-Cortical Brain Organisation Shapes the Neural Representation of Painful Symptoms in Late Adolescence.** Related to Figure 1 and 2. Associations (β) of rsFC with painful symptoms at TP1 (lower left) and TP2 (upper right). Significant associations ($p_{adj}$ ≤.05) are highlighted. Names of ROIs with >3 significant results are written out. RH = right hemisphere, LH= left hemisphere.
